# Preeclampsia is associated with increased maternal body weight in a northeastern Brazilian population

**DOI:** 10.1186/1471-2393-13-159

**Published:** 2013-08-08

**Authors:** Edailna Maria de Melo Dantas, Flávio Venicio Marinho Pereira, José Wilton Queiroz, Diogo Luis de Melo Dantas, Gloria Regina Gois Monteiro, Priya Duggal, Maria de Fatima Azevedo, Selma Maria Bezerra Jeronimo, Ana Cristina Pinheiro Fernandes Araújo

**Affiliations:** 1 Health Sciences Post-Graduate Program, Natal, RN, Brazil; 2 Institute of Tropical Medicine of Rio Grande do Norte, Natal, RN, Brazil; 3 Department of Biochemistry, Biosciences Center, Federal University of Rio Grande do Norte, Natal, Rio Grande do Norte, Natal, RN, Brazil; 4 Institute of Science and Technology of Tropical Diseases (INCT-DT), Natal, RN, Brazil; 5 Department of Epidemiology, Bloomberg School of Public Health, Johns Hopkins University, Baltimore, MD, USA; 6 Department of Internal Medicine, Health Science Center, UFRN, Natal, RN, Brazil; 7 Department of Obstetrics and Gynecology, Health Science Center, UFRN, Natal, RN, Brazil

**Keywords:** Preeclampsia, BMI, Obesity, Hypertension, Pregnancy

## Abstract

**Background:**

Preeclampsia is a disease with great variability in incidence across the world. The mortality is higher in lower income countries, where it is the leading cause of maternal mortality. This study aimed to determine the frequency of and risk factors for preeclampsia in a low income population from an urban area of Brazil.

**Methods:**

A prospective case control study of 242 women of which 30 developed preeclampsia, 4 had gestational hypertension, 2 had superimposed hypertension, 11 had spontaneous abortion, 13 were lost to follow up and 192 had normal pregnancy. This latter group was considered the normotensive controls. The rate of preeclampsia and the risk of cardiovascular disease, after onset of preeclampsia, were determined.

**Results:**

Of the 218 women who completed the study, the frequency of hypertensive disorder of pregnancy was 16.5% (36 of 218) and of preeclampsia was 13.8% (30 of 218). Women with preeclampsia had a higher body mass index (BMI), mean of 25.3 ± 4.8 compared to 23.5 ± 3.7 for the normotensive controls, p = 0.02. The risk of preeclampsia increased with BMI [Odds ratio (OR) 1.12, 95% Confidence Interval (CI = 1.02;1.24, p-value = 0.023)]. Women with preeclampsia developed chronic hypertension more often than normotensive controls (p = 0.043) and their systolic and ambulatory blood pressure monitoring was elevated (p = 0.034). Women with preeclampsia had higher BMI even 5 years post-pregnancy (p = 0.008).

**Conclusions:**

Women who are overweight or older have an increased risk of preeclampsia. Previous history of preeclampsia increases the risk of early onset of chronic hypertension. Therefore, effective preventive measures are needed, particularly women at lower social economic stratum who have less access to proper medical care and adequate nutrition.

## Background

Preeclampsia is a heterogeneous disease unique to humans and is the leading cause of maternal and intrauterine mortalities [1,2] and intrauterine growth restriction [3]. Because of maternal complications, there is usually a need for early interruption of the pregnancy. Preeclampsia increases the risk of maternal mortality in both developed (1.8%) and less developed countries (14%) [4-6] and it is the leading cause of morbidity and mortality of women of child bearing age worldwide [7,8]. Several factors including ethnicity, obesity, metabolic syndrome, excessive weight gain, multiparity, chronic hypertension, diabetes mellitus, and thrombophilias increase the risk of preeclampsia [1,9]. Socio-economics also plays a role in the vulnerability of women to develop preeclampsia. Women with lower income have more difficulty in access to medical care, early diagnosis and proper screening and therapy, and they often have inadequate nutrition [10-12].

The principal cause of maternal mortality in Brazil is preeclampsia. This has been fostered by an increasing prevalence of obesity, hypertension and insulin resistance [13-15], along with an increasing incidence of chronic diseases like cardiovascular disease, diabetes, and auto-immune disorders [16]. Although there is universal health care in Brazil, there is still a need for improvement, principally with respect to women’s health [17].

Studies to date have determined preeclampsia rates by hospital discharge summaries or national registries [18-20]. But a limitation of this approach is the bias of those localities using a reference center in which the reported cases are more severe. However, to our knowledge there is no study evaluating the community based incidence of preeclampsia in Brazil or in low economy countries. In this study, we aimed to determine the rate of preeclampsia by recruiting pregnant women at their first medical visit in a community health post center in Natal, Brazil. We also evaluated their hypertensive status 5 years after the onset of preeclampsia.

## Methods

### Study design

A prospective study of pregnancy outcomes was conducted from August 2004 to July 2006 in the district of Bom Pastor, Natal, Rio Grande do Norte, northeast Brazil to determine the incidence of preeclampsia. The district of Bom Pastor is located in the western region of Natal, with a population of 10,933 people of which 52% are female. In the district, 64.9% of women were of childbearing age (n = 3718), and of these 6.5% (n = 242) became pregnant during the study period. A total of 242 women were recruited, and the mean gestational age at enrollment was 18.4 ± 7.9 weeks. Eleven women (4.5%) had spontaneous abortion and 13 (5.4%) were lost to follow up, resulting in 218 women who were followed through the end of the pregnancy. Of these 218 women, 30 developed preeclampsia (13.7%), 4 had gestational hypertension (1.8%) and 2 (0.9%) had superimposed hypertension in pregnancy. In this study, we compared cases of preeclampsia to those who had a normotensive pregnancy (n = 182), (Table 1).

**Table 1:** Clinical and obstetric characteristics of pregnant women in a community based study

**Variables**	**Preeclampsia (n = 30)**	**Normotensive controls (n = 182)**	**p-value**^**(1)**^
Maternal age (years)	26.9 ± 7.9	23.9 ± 5.8	0.014
Number of gestations	2.3 ± 3.0	2.5 ± 1.7	0.647
Mean Parity	1.1 ± 2.9	1.2 ± 1.3	0.873
BMI at recruitment (kg/m^2^)	25.3 ± 3.9	23.7 ± 3.6	0.020
Gestational age at recruitment (weeks)	19.6 ± 8.2	18.7 ± 7.8	0.599
Gestational age at delivery (weeks)	37.6 ± 3.2	39.1 ± 1.9	0.015
Maternal Initial weight (kg)	63.9 ± 11.2	58.3 ± 9.3	0.038
Gestational age at delivery (weeks)	37.6 ± 3.2	39.1 ± 1.9	0.015
Infant birth weight (g)	3028 ± 802	3236 ± 491	0.178
AST (U/l)	16.6 ± 4.1	15.2 ± 5.1	0.193
ALT (U/l)	14.8 ± 5.7	11.9 ± 5.5	0.012
Total Cholesterol (mg/dl)	227.1 ± 57.2	221.2 ± 54.2	0.607
Tryglicerides (mg/dl)	190.1 ± 61.8	177.8 ± 59.5	0.326
HDL (mg/dl)	49.1 ± 13.6	48.9 ± 11.4	0.964
LDL (mg/dl)	139.3 ± 53.8	136.2 ± 46.7	0.762
VLDL (mg/dl)	37.5 ± 12.4	35.1 ± 11.9	0.343
Glucose (mg/dl)	64.9 ± 11.0	68.4 ± 47.6	0.714
Urea (mg/dl)	20.7 ± 4.1	22.0 ± 5.6	0.272
Creatinine (mg/dl)	0.7 ± 1.4	0.2 ± 0.8	0.006
UricAcid (mg/dl)	3.1 ± 1.4	2.9 ± 1.3	0.598
Family history of chronic hypertension			0.949
Yes	17 (56.7%)	102 (56.0%)	
No	13 (43.3%)	80 (42.0%)	
Family history of preeclampsia (mother)			0.314
Yes	2 (6.7%)	5 (2.7%)	
No	28 (93.3%)	177 (97.3%)	
Family history of preeclampsia (sibling)			0.618
Yes	2 (6.7%)	7 (3.8%)	
No	28 (93.3%)	175 (96.2%)	
Type of delivery			<0.001
Vaginal	8 (26.7%)	141 (77.5%)	
Cesarean section	22 (76.3%)	41 (22.5%)	
Age group			0.087
< 20 years	4 (13.3%)	48 (26.4%)	
20 – 24 years	8 (26.7%)	61 (35.3%)	
25 – 34 years	12 (40.0%)	60 (33.0%)	
> 34 years	6 (20.0%)	13 (7.1%)	
BMI by interval (kg/m^2^)			0.070
< 18.5	1 (3.3%)	12 (6.6%)	
18.5 – 24.9	11 (36.7%)	107 (58.8%)	
25.0 – 29.0	14 (46.6%)	52 (28.6%)	
> 29.0	4 (13.3%)	11 (6.0%)	

(1) Student – t test for continue variables or Chi-Square test for categorical variables, data mean ± Sd.

The household income for this population was 2.2 monthly Brazilian minimal wages (~US$ 440 monthly), (http://www.ibge.gov.br). Approximately 22% of the population had no schooling and 22% had an average of 3 years of education. The overall literacy rate for women was 55.9% (http://www.ibge.gov.br; http://www.natal.rn.gov.br/semurb/paginas/ctd-106.html).

Each subject was interviewed and examined by the same physician (EDM). Personal and family histories of diseases were determined. Anthropometric measurements were recorded at each visit. Blood was collected each trimester to assess complete blood count, liver function, lipid profile, and venereal disease (syphilis). HIV status was checked once during pregnancy. Urinalysis was completed each trimester. Since the goal was to determine the profile of pregnancy outcomes in a community health center there were no exclusion criteria.

A follow up study in a subgroup of women (n = 27) was performed five years after the initial recruitment. Of this group, 10 had preeclampsia and 17 had no history of preeclampsia. Ambulatory blood pressure monitoring was measured. Preeclampsia was defined by the Report of the National High Blood Pressure Education Program [21].

### Ethical considerations

The protocol was reviewed and approved by the Federal University of Rio Grande do Norte Ethical Committee (CEP-UFRN 89–02) and the Brazilian National Ethical Research Committee (Comissão Nacional de Ética em Pesquisa- CONEP/MS 5060), with an amendment (CEP-UFRN 006–2011), all participants signed the informed consent.

### Statistical analysis

The mean of continuous variables such as age and blood pressure were compared between the groups using the Student t-test. Qualitative variables such as presence of complications were analyzed by contingency tables using the Maximum Likelihood test to assess the hypothesis of association. Box plots were used to show the distribution of BMI. To estimate the effect of age and BMI as a risk for preeclampsia, a logistic regression model used age and BMI as predictors variables to determine the relative risk (RR) of preeclampsia using the formula: log(*RR*) = *β*_0_ + *β*_1_*AGE* + *β*_2_*BMI* + *error*. The hypothesis of no association *H*_0_:*β*_*i*_ = 0 was rejected when the p value was less than 0.05. Comparisons of the continuous variable’s distributions assessed only in the second moment used the Mann–Whitney test, (p < 0.05). Parameters measured after delivery included BMI, parity, total cholesterol, HDL, triglycerides, glucose, urea, creatinine, microalbuminuria and Ambulatory Blood Pressure Monitoring (ABPM), which were analyzed as continuous variables. ABPM was determined in accordance with the International Academy of Cardiology [22,23]. Cluster analysis of the districts in Natal was analyzed considering both income distribution and literacy rate. A Hierarchical Cluster with the Ward’s Method using the Squared Euclidian Distance was performed to determine the dissimilarity among the districts.

## Results

**Figure 1:**
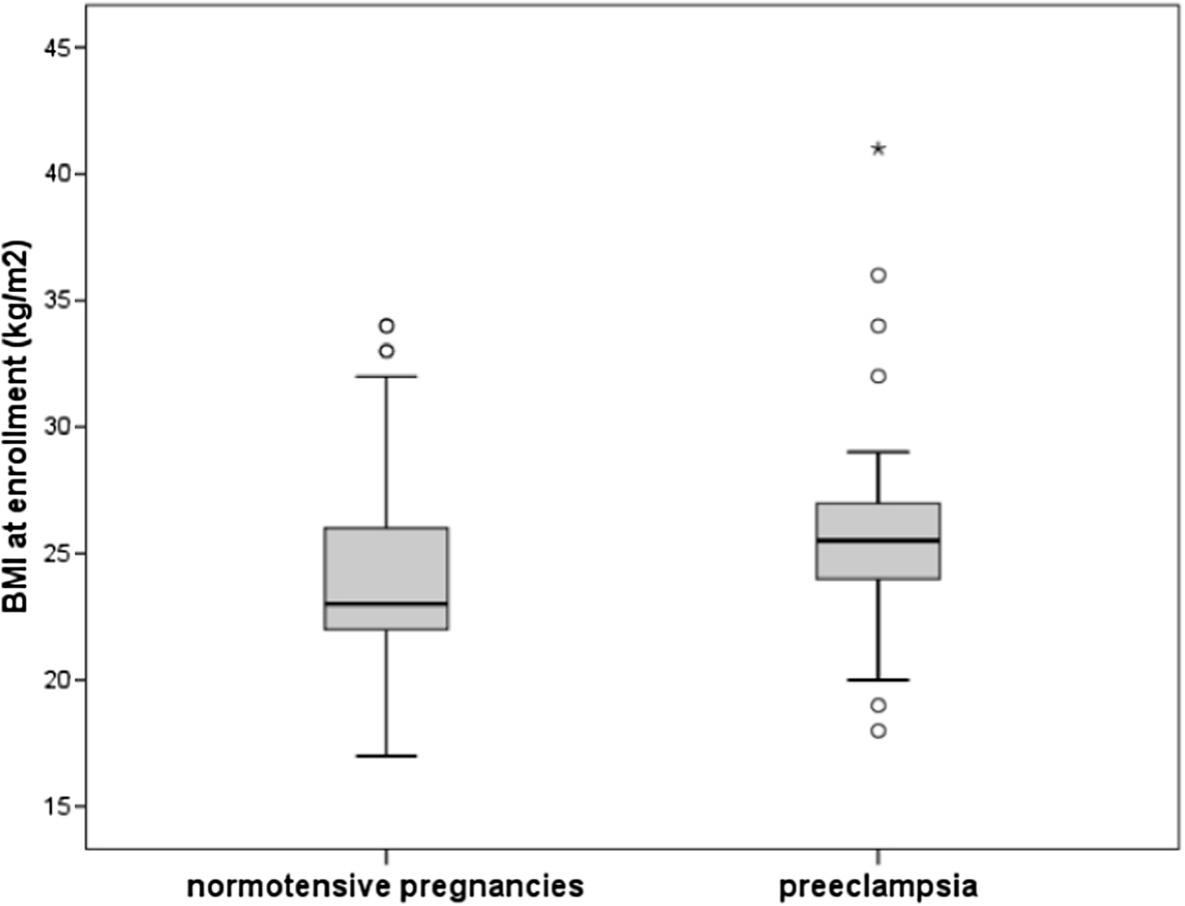
Comparison of body mass index (BMI) between women who developed hypertensive disorder or normal pregnancy.

Of the 212 women who were studied until the end of the pregnancy, 30 had preeclampsia. Of those who developed preeclampsia, 20% (6 of 30) had severe preeclampsia and received anti-seizure therapy, and one woman developed HELLP syndrome. There was no difference in the number of prior gestations between the women who developed preeclampsia compared to the normotensive controls, 2.3 ± 3.0 and 2.5 ± 1.7 (p = 0.647), respectively, nor in the gestational age at recruitment, 19.6 ± 8.2 and 18.7 ± 7.8 weeks (p = 0.599), respectively, (Table 1). However, women who developed preeclampsia had a higher weight at enrollment than the normotensive controls, (63.9 ± 11.2 kg vs. 58.5 ± 9.3 kg, p = 0.038), (Table 1). The BMI at recruitment was also higher among women who developed preeclampsia (25.3 ± 3.9 vs. 23.7 ± 3.6 kg/m^2^, p = 0.020), Figure 1. Women with preeclampsia also had higher creatinine 0.7 ± 1.4 vs. 0.2 ± 0.8 mg/dl (p = 0.006) (Table 1).

**Figure 2:**
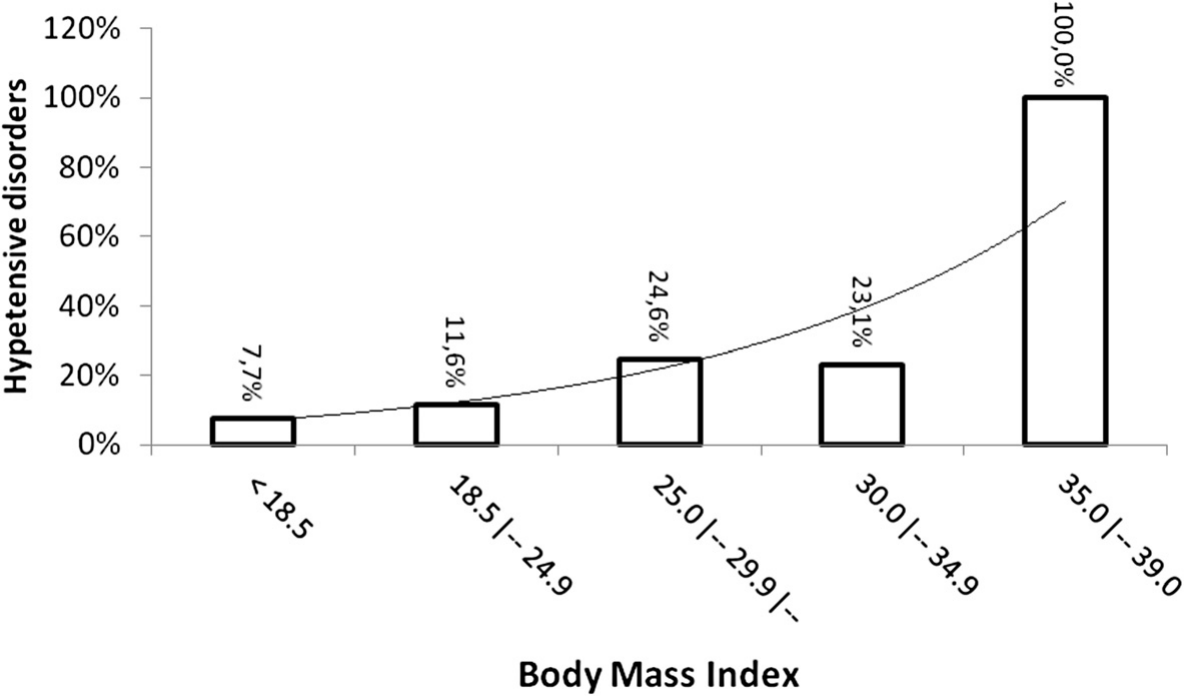
**The percent of women with hypertensive disorder of pregnancy versus age.** X axis represents age and Y axis represents the percent of women with hypertensive disorder of pregnancy within that age group.

**Figure 3:**
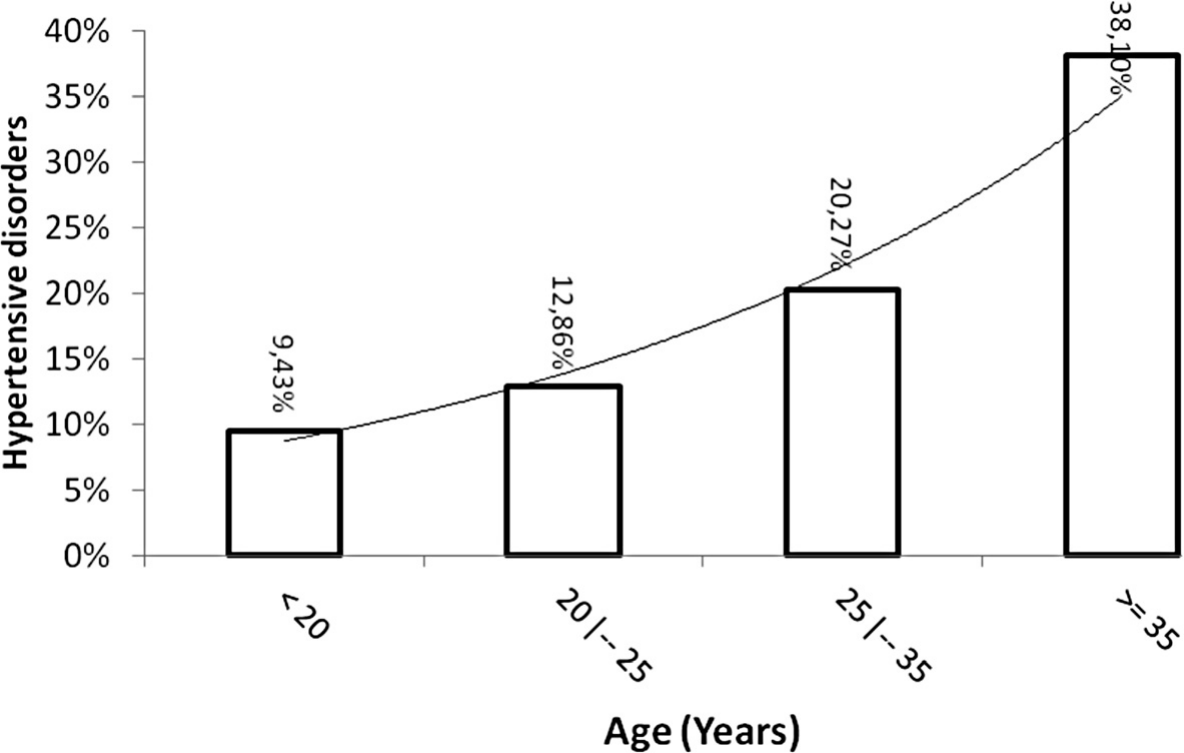
**The percent of women with hypertensive disorder of pregnancy versus body mass index.** X axis represents BMI by interval and Y axis represents the percent of women with hypertensive disorder of pregnancy per interval of BMI.

No association with family history of hypertension or preeclampsia was observed (Table 1). The risk of preeclampsia increased exponentially with age, (6% increase risk per year, p = 0.090), and as represented in Figure 2, and with BMI (12% increase risk for each unit of increased BMI, p = 0.023), determined by using a binary logistic model corrected by age and BMI, (Table 2), Figure 3. The level of schooling was lower in women with preeclampsia, with only 20% having completed high school (p = 0.025).

**Table 2:** The relative risk of preeclampsia considering age and body mass index

**Variable**	**Estimate**	**Odds-ratio**	**95% conf. interval**	**p value**
Intercept	β_0_ = - 5.982	-		0.0001
AGE	β_1_ = 0.053	1.06	0.99; 1.12	0.090
BMI	β_2_ = 0.114	1.12	1.02; 1.24	0.023

There was no difference in the weight of the newborn between children born of preeclampsia and normotensive controls pregnancies (p > 0.05). However, children born of a preeclampsia pregnancy had a lower APGAR at birth (p = 0.014), but with a significant recovery at 5 minutes APGAR (p = 0.039), Table 3.

**Table 3:** Characteristics of the children at birth

**Children outcome at birth**	**Preeclampsia n = 30**	**Normotensive controls n = 182**	**p-value**
Weight (g)	3029 ± 800	3236 ± 490	0.180
APGAR 1*	7.5 ± 2.0	8.2 ± 1.0	0.014
APGAR 5*	8.7 ± 1.0	8.9 ± 1.0	>0.050

*The APGAR 1 and 5 for children born of preeclampsia was different (Student T-Test, p = 0.039), data mean ± Sd.

A subset of subjects which included 10 cases and 17 controls was followed 5 years post-delivery. BMI at 5-year follow up were still higher among women who had prior preeclampsia (28.5 ± 1.5 vs. 25.0 ± 2.8 kg/m^2^, p = 0.013). Half of the follow up group with prior preeclampsia (5/10) developed chronic hypertension while only 11.8% (2/17) did in this with prior normotensive pregnancy (p = 0.040) (Table 4). All hypertensive cases were being treated. Even so, women with prior preeclampsia had significantly higher ABPM 24 hour diastolic blood pressure (p = 0.017) as well as ABPM awake-Diastolic blood pressure (p = 0.010) when compared to the normotensive controls (Table 4). There was no difference in the growth of the child when considering the type of pregnancy and the gestational age at delivery (Table 5).

**Table 4:** Metabolic parameters in women with a history of preeclampsia or normotensive controls were evaluated prior to delivery and at five years follow up

**Variables**	**Median ± half interquartile range**		**p-value**^**(1)**^
	**Preeclampsia (n = 10)**	**Normotensive controls (n = 17)**	
**Variables at recruitment**			
Age (years)	27.0 ± 6.7	26.0 ± 2.5	0.414
BMI (kg/m^2^)	26.5 ± 1.5	23.6 ± 3.9	0.170
Parity	0.5 ± 1.0	1.0 ± 1.0	0.204
Total Cholesterol (mg/dl)	226.0 ± 65.5	246.0 ± 36.7	0.426
Triglycerides (mg/dl)	163.0 ± 51.2	217.0 ± 49.5	0.181
HDL (mg/dl)	50.0 ± 7.7	45.0 ± 5.2	0.263
Ratio Cholesterol-HDL	4.4 ± 1.6	5.5 ± 1.3	0.241
Glucose (mg/dl)	65.0 ± 4.5	64.0 ± 7.0	0.711
Urea (mg/dl)	21.0 ± 2.9	19.0 ± 2.4	0.133
Creatinine (mg/dl)	0.8 ± 0.1	0.7 ± 0.1	0.051
Uricacid (mg/dl)	3.1 ± 1.5	3.7 ± 1.2	0.833
**At 5 year follow up**			
BMI (kg/m^2^)	28.5 ± 1.5	25.0 ± 2.7	0.013
Abdominal circunference (cm)	91.5 ± 9.4	85.0 ± 6.8	0.127
Parity	2.0 ± 0.7	3.0 ± 1.0	0.386
Total cholesterol (mg/dl)	180.5 ± 11.6	173.0 ± 28.8	0.537
Triglycerides (mg/dl)	85.5 ± 33.2	114.0 ± 39.8	0.675
HDL (mg/dl)	48.5 ± 7.0	52.0 ± 5.8	0.604
RatioCholesterol-HDL	3.7 ± 0.4	3.3 ± 0.7	0.414
Glucose (mg/dl)	76.0 ± 7.6	76.0 ± 4.8	0.473
Urea (mg/dl)	20.0 ± 3.6	20.0 ± 3.5	0.570
Creatinine (mg/dl)	0.7 ± 0.1	0.7 ± 0.2	0.386
Uricacid (mg/dl)	3.3 ± 0.4	3.2 ± 0.8	0.414
RatioAlbumin/creatinine	31.9 ± 18.8	24.5 ± 16.6	0.414
C reactiveProtein (mg/dl)	0.1 ± 0.2	0.1 ± 0.1	0.824
Ambulatory blood pressure monitoring (ABPM - mmHg)			
24 hour systolic Blood Pressure	113.0 ± 6.7	104.0 ± 7.2	0.053
24 hour Diastolic Blood Pressure	75.5 ± 6.9	67.5 ± 5.1	0.017
Daytime Systolic Blood Pressure	116.0 ± 7.1	108.5 ± 7.9	0.097
Daytime Diastolic Blood Pressure	80.5 ± 6.5	72.0 ± 6.0	0.010
Sleep Systolic Blood Pressure	108.5 ± 4.9	97.5 ± 8.6	0.053
Sleep time Diastolic Blood Pressure	67.0 ± 4.2	61.0 ± 5.1	0.097

(1) Median test for independent groups.

**Table 5:** Weight of the children at birth and at follow up

**Time**	**Children born of**		**p-values**
	**Preeclampsia n = 10 (mean ± SD)**	**Normotensive controls n = 17 (mean ± SD)**	
Gestational age at delivery (weeks)	38.9 ± 3.6	38.4 ± 2.1	0.667
Birth (g)	3151 ± 780	3081 ± 476	0.388
1 month (g)	4042 ± 1115	4313 ± 708	0.447
2 months (g)	5813 ± 1062	6122 ± 864	0.392
3 months (g)	7473 ± 1796	7782 ± 968	0.640

Because of the high rate of preeclampsia observed, we analyzed whether socio-economics or literacy were similar in other districts in Natal and other localities in Brazil. Cluster analysis of the district showed that there were five clusters in Natal, and the studied district fell into the cluster with high population density and lower socioeconomics (Additional file 1: Table S1). In general, women in the lower social economic stratum are more vulnerable with respect to access to medical care, adherence to treatment and adequate nutrition.

## Discussion

Hypertensive disorders of pregnancy have a high incidence worldwide. Over 500,000 women die yearly due to causes related to pregnancy and the majority of these deaths occur in less developed countries [12,24]. In addition, children born of a preeclampsia pregnancy have an increased risk of endocrine, nutritional, metabolic, and hematopoietic diseases [25-27]. Anecdotal reports indicate that all hypertensive disorders of pregnancy are high in Brazil, although there is no epidemiological study showing the actual frequency of preeclampsia in Brazil. In this Brazilian cohort, the incidence of hypertensive disorders of pregnancy was 16.9% and the frequency of preeclampsia was 13.8%. We anticipate that the rates of preeclampsia in other underserved areas may also be higher than expected [28].

The cause of preeclampsia is unknown, but previous studies have found an association of elevated BMI and preeclampsia [29]. Women with lower body mass index (BMI) have a lower risk of preeclampsia than women at normal or higher BMI [6,30,31]. The mechanism by which obesity increases the incidence of preeclampsia could include increased insulin resistance or inflammation [32,33]. Insulin resistance has been associated with endothelial dysfunction [14,15], and increased secretion of endothelin 1, a potent vasoconstrictor [34]. In addition, insulin resistance results in reduction of nitric oxide, increasing the risk of hypertension and cardiovascular diseases [20,35-40]. Association of higher BMI and preeclampsia was also seen in our cohort, although the mean BMIs for our study population are not those of severe obesity. Women of Latin American origin showed a strong association with higher BMI and preeclampsia [41,42]. These changes may be the result of the socio-economic changes that have occurred in Brazil with increased urbanization and substantial dietary changes with increased availability of food [43,44].

In addition to obesity, others have hypothesized that preeclampsia could be related to the inflammatory response induced by infectious agents. Studies have shown that women with periodontal disease have increased risk of preeclampsia [10,17]. A metanalysis of observational studies indicated that the risk of preeclampsia was increased in pregnant women with urinary tract infection and periodontal disease [10,17,45,46]. Overall, there is poor dental health in this population and the majority of women have gingivitis and dental cavities (personal observation, SMBJ), although these issues were not assessed in this study.

The World Health Organization indicates that the frequency of cesarean section is ~15% worldwide [47], with the rate in Africa being about 4% [38], and 30% in the United States, [48,49]. Overall the rate of C section is high in Brazil, and even higher in women who have access to private health insurance, with rates greater than 50% [50,51]. But preeclampsia is a condition that may warrant a cesarean delivery [52], because the need to save the child and to avoid greater complications of the preeclampsia, including eclampsia. Although the use of magnesium sulfate has decreased mortality and morbidity [53], the severity of the preeclampsia leads to resolution of the pregnancy with indication of cesarean section as a mean to decrease the fetal and maternal complications [48,54]. In this study, the frequency of cesarean section in women with normal pregnancy was 22%, compared with 60% in women with preeclampsia. In a previous, study of a similar population of women with lower income; the rate of cesarean deliveries with severe preeclampsia was 80%, whereas the rate of cesarean section in the normotensive controls group was 20% [55].

Children born of a preeclampsia pregnancy have more long term complications [25,26]. In addition, the impact of the inflammation due to the preeclampsia status on the fetus is still not fully understood. At one year post-partum, the subset of children born to this cohort showed normal growth and gain of weight. This outcome may have been influenced by the careful monitoring of the mother during the pregnancy, since all women who develop preeclampsia were enrolled promptly into therapy; different from our findings from a referral center in which we observed more severe cases with the majority of them without adequate care during the pregnancy [55]. However, additional term follow up is needed to assess long-term outcomes for children of women with preeclampsia.

A follow up of a subgroup of the study population revealed that 50% of the women who had preeclampsia were still hypertensive 5 years after delivery. Their BMI was significantly higher than women who had a non-hypertensive pregnancy. Increased body weight is a potentially modifiable risk factor; controlling weight could help reduce the risk of maternal complications such as preeclampsia. In addition, there is a need for careful post-partum follow-up to better manage hypertension and to counsel regarding BMI. Although we did not find an increased risk of preeclampsia with family history of chronic hypertension or preeclampsia, as we had previously observed, (55) this could be because most of these cases were considered mild, except 6 preeclampsia cases that needed magnesium sulfate and 1 HELLP syndrome case. In summary, we found that women who are underserved in terms of health care had a higher frequency of preeclampsia and that preeclampsia is a frequent disease in this population. In addition, higher BMI and a hypertensive condition may contribute to earlier onset of cardiovascular disease. As obesity and diabetes continue to rise in prevalence in countries like Brazil, more attention post-delivery is warranted to decrease long term complications.

## Conclusions

Higher BMI and older age during the pregnancy were associated with hypertension. History of preeclampsia increases the risk of early onset of chronic hypertension.

## Competing interest

The authors declare that they have no competing interests.

## Authors’ contributions

EM wrote the proposal, participated in data collection, analyzed the data and drafted the manuscript. FVMP, GRM, DM and MFZ participated in data collection and revised the manuscript; JWQ performed data analysis and revised the manuscript; PD drafted and revised the manuscript. SMBJ and ACA approved the proposal with some revisions, participated in data analysis and revised subsequent drafts of the paper. All authors read and approved the final version of the manuscript and approve its submission.

## Authors’ information

EM is an Obstetrician working with a team of researchers focusing on understanding the risk factors to developing Preeclampsia. These data set the stage to estimate the risk of preeclampsia in the context of changes in the disease pattern in Brazil and at the same time is helping to tease out the influence of genetics versus environment in the risk of developing preeclampsia and its complications.

## Pre-publication history

The pre-publication history for this paper can be accessed here:

http://www.biomedcentral.com/1471-2393/13/159/prepub

## Supplementary Material

Additional file 1: Table S1 Cluster analysis of population density, income and literacy for the Natal Districts. (*) Based in the 2010 census, which showed that population was 801,164 people. Note that Bom Pastor District belong to cluster one. Click here for file
