# Supplementary material for: Preeclampsia is associated with increased maternal body weight in a northeastern Brazilian population
Source: BMC Pregnancy Childbirth. 2013 Aug 8;13:159. doi: 10.1186/1471-2393-13-159 (PMC4231463; doi:10.1186/1471-2393-13-159)
Supplement: Additional file 1: Table S1 — Cluster analysis of population density, income and literacy for the Natal Districts. (*) Based in the 2010 census, which showed that population was 801,164 people. Note that Bom Pastor District belong to cluster one. [file 1471-2393-13-159-S1.doc]

Supplemental Table 1. Cluster analysis of population density, income and literacy for the Natal Districts.

| Cluster Number | Income | | % of Literacy | % of Population |
| --- | --- | --- | --- | --- |
|  | % < 1 Minimal wage | % > 10 Minimal wage |  |  |
| 1 | 57.5 | 0.5 | 88.8 | 54.6 |
| 2 | 42.3 | 3.4 | 93.8 | 20.3 |
| 3 | 24.4 | 10.2 | 96.9 | 9.4 |
| 4 | 17.4 | 20.7 | 98.2 | 14.2 |
| 5 | 83.3 | 0.1 | 77.6 | 1.4 |

(*) Based in the 2010 census, which showed that population was 801,164 people. Note that Bom Pastor District belong to cluster one.
